# Altered microbiome and metabolome profiling in fearful companion dogs: An exploratory study

**DOI:** 10.1371/journal.pone.0315374

**Published:** 2025-01-15

**Authors:** Luigi Sacchettino, Michele Costanzo, Iolanda Veneruso, Valeria D’Argenio, Maria Mayer, Francesco Napolitano, Danila d’Angelo

**Affiliations:** 1 Department of Veterinary Medicine and Animal Production, University of Naples Federico II, Naples, Italy; 2 Department of Molecular Medicine and Medical Biotechnology, University of Naples Federico II, Naples, Italy; 3 CEINGE-Biotecnologie Avanzate Franco Salvatore, Naples, Italy; 4 Department of Human Sciences and Quality of Life Promotion, San Raffaele Open University, Rome, Italy; 5 Independent Researcher, Small Animal Nutrition Consultation (FNOVI), Rome, Italy; University of Wisconsin-La Crosse, UNITED STATES OF AMERICA

## Abstract

Behavioral dysfunctions in dogs represent one of the main social concerns, since they can endanger animals and human-dog relationship. Together with the trigger stimulus (human, animal, place, scent, auditory stimuli, objects), dogs can experience stressful conditions, either in multiple settings or unique situations, more often turning into generalized fear. Such a dysfunctional behavior can be associated with genetic susceptibility, environmental factors, traumatic experiences, and medical conditions. The available therapy, based on behavior approaches, environmental management, and neurochemical manipulation, through nutrition, supplements, medicines, and pheromones, represent the mainstays of the treatments currently accessible. Growing evidence in humans and animals highlight the importance of the gut-brain axis in the modulation of the brain physiology and behavior as well. Here, taking advantage of the next generation sequencing approach, we sought to investigate the potential connection between gut microbiota and microbiome in dogs suffering from generalized fear (n = 8), when compared to healthy subjects (n = 8), who all lived in different families. Faecal microbiota evaluation showed a differential abundance of taxa related to Proteobacteria and Firmicutes Phyla, between case and control dogs. Moreover, serum metabolomics documented significant alterations of molecules associated to GABA and glutamate neurotransmission in the patients, as well as bile acids metabolism. Overall, our preliminary and integrated investigations highlighted an intriguing role for the microbiome-metabolome network, allowing to further unveil the potential pathophysiology of relational issues in companion animals and paving the way for more effective therapeutical approaches.

## Introduction

In mammals, the gastrointestinal (GI) tract is populated by a huge variety of symbiotic microorganisms, acquired from maternal microbiota during pregnancy, and influenced by mothers’ diet and delivery methods [1]. Accordingly, studies in 91 pregnant women showed a significant increase of *Enterococcus*, alongside with lower levels of both *Enterobacter* and *Streptococcus* during the early phases of gestation [2]. Moreover, bacteria belonging to Bacteroidetes and Actinobacteria phyla are precociously transmitted to infants through the natural childbirth. On the other hand, caesarean section or antibiotic exposure around birth were found to be causative of a higher incidence in pathogen colonisation and immune-related disorders in children [3]. In general, healthy dogs’ microbiomes show more similarities to humans than cats [4], likely due to a shared environment and a relevant starch-enriched diet [5–9]. Firmicutes, Bacteroidetes, Proteobacteria, Fusobacteria, and Actinobacteria phyla build up more than 99% of all intestinal bacteria in dogs [9, 10], while at the genus level, the GI microbiota has been considered dominated by *Fusobacterium*, *Bacteroides*, and *Prevotella* [4, 7, 11]. Studies about GI microbiota and its biogeography in mouse models documented the spatial distribution of specific phyla, which are differentially localized between fore and hindgut, depending on chemical gradients, nutrition availability and intestine immunity [12]. In keeping with that, the stomach harbours an acid resistant flora of aerobic and anaerobic bacteria, populated by *Helicobacter*, *Lactobacillus*, *Streptococcus* and *Clostridium* [13]. On the other hand, facultative aerobic or anaerobic microorganisms, including *Clostridia*, *Lactobacillus* and Proteobacteria inhabit small intestine, whereas the large intestine of both cats and dogs host anaerobic bacteria, such as *Clostridiales*, *Bacteroides*, *Prevotella* and *Fusobacteria* [10, 14, 15]. Research studies about GI microbiota in both humans and animals often do not account for the microbiota diversity within specific sections of the gut, thus representing a generalized profiling of the whole GI tract. In this framework, canine faecal samples could nevertheless provide a proper snapshot of the most relevant gut microbiota taxa composition compared to humans, since they have a more rapid transit time, with a shorter stay within mucosa membranes [16, 17]. In mammals, GI microbiota profiling can be modulated by several factors, such as sex, age, breed, diet, gastrointestinal disease, body condition score (BCS), reproductive status and environment, according to different species [11, 14, 16, 18–24]. Moreover, several pharmacological treatments with pump inhibitors, antidepressants, glucocorticoids, or antibiotics can even have detrimental impact upon mammalian GI microbiota [25–30]. Besides its crucial role in digestion, several reports highlighted the importance of the GI microbiota in integrating endocrine, immune, and neural pathways. In this respect, the bidirectional activity of the gut-brain-immune axis allows the brain to modulate the GI activity, whereas GI tract itself can shape CNS functioning. According to human and veterinary medicine studies, disruption of the gut-brain- axis homeostasis can be associated with the onset and severity of behavioral disorders [31–35]. Such a mutual communication system is exploited by the ability of the microbiota to synthetise specific metabolites and neurotransmitters, which might influence mood-related behaviors, alongside with emotion, motivation, reward and cognition [36–42]. Metabolome is defined as the complete set of low-molecular weight molecules occurring in a peculiar biological system, thus allowing to draw a metabolic fingerprinting that may reflect systemic changes [43, 44]. Hence, the intricate metabolic interactions taking place between gut microorganisms and their hosts might be addressed through the investigation of a metabolome profiling [45]. Indeed, the communication between brain, microbe communities and gut have already been widely assessed as continuous and bidirectional [46]. It is not surprising that irritable bowel syndrome (IBS) and inflammatory bowel disease (IBD) were linked to changes in the microbiota-gut-brain axis [47, 48]. Additionally, these intestinal disorders appeared to be associated with mood impairments, such as anxiety, depression [49]. Therefore, questioning whether the microbiota modulates dog’s behavior and vice versa will be helpful. Kirchoff and colleagues (2019) found a clustering of faecal taxa in 21 dogs with aggressive conspecific behavior, thus assuming that gut microbiome profiling might be considered a useful tool for a diagnostic assessment of aggressive behaviors [50]. Further investigation in a small sample of phobic, aggressive, and anxious dogs showed a different structure and composition of the gut microbiota in dogs with behavioral issues, when compared to healthy controls [51, 52]. Therefore, in the present work we sought to examine the potential impact of GI microbiota, together with a serum metabolomic profiling, in fearful family dogs.

## Methods

### Ethics statement

All experimental protocols were approved by the Scientific Ethic Committee for Animal Experimentation (Reference number: PG/2023/0011527), in accordance with the Italian legislative Decree (N. 26/ 2014). The blood samples were taken during routine visits, as a part of screening for health problems. Written informed consent was obtained from each owner for the publication.

### Animals and sample collection

Fearful (case) dogs and healthy (control) subjects (n = 8) were between one and eight years old, clustered for breed, sex, age, and weight (Table 1). Both patients and controls were owned dogs, fed with dry commercial diet containing crude protein (24–28% of total content), fat (11–18%), fiber (2,2–14%), ash (5,8–11%), and moisture (8–9%). All the enrolled subjects lived in different Italian cities, namely Caserta, Naples, Salerno (Campania Region) and Formia (Lazio Region), and did not receive antibiotics four weeks before stool and blood sampling. Neither glucocorticoid, proton pump inhibitors and/or probiotics, herbal remedies and nutraceuticals were provided. One of the behaviorist veterinarians carried out clinical and behavioral examination for each animal, who then categorized according to their behavioral phenotype (fearful vs control group). Diagnostic clues of fear were hypervigilance, scanning, changes in social soliciting behavior (hiding, escape attempts), displacement behaviors, out-of-context grooming and scratching, yawning, lip licking, whining; physiologic signs (trembling, dilated pupils, hypersalivation, tachypnoea, tachycardia). The behavioural history included inadequate early environmental experiences and/or inadequate socialisation. All dogs were subjected to physical examinations by a licensed veterinarian, including recording their body weight and body condition score (using a 9-point scale: underweight (1–3), ideal (4–5), and overweight (6–9)).

**Table 1:** Demographics of the dogs enrolled in the study.

Subject/Breed	Group	Sex	Age (years)	Reproductive status	Weight (Kg)	BCS (1–9 scale)	City (Region)
Monky/Mongrel	Case	Male	2	Neutered	18	4	Caserta (Campania)
Mira/Pinscher mix-breed	Control	Female	3	Spayed	5	5	Caserta (Campania)
Cam/Weimaraner	Control	Male	4	Intact	38	5	Salerno (Campania)
Macchia/Mongrel	Case	Female	2	Spayed	14	4	Salerno (Campania)
Ciammarica/Mongrel	Case	Female	5	Spayed	6	4	Salerno (Campania)
Otto/Rottweiler	Control	Male	2.5	Intact	50	5	Salerno (Campania)
Rena/Mongrel	Case	Female	1.5	Spayed	30	4	Formia (Lazio)
Charlotte/Mongrel	Case	Female	7	Spayed	7	5	Formia (Lazio)
Maya/Mongrel	Case	Female	2	Spayed	20	5	Formia (Lazio)
Rosina Mongrel	Case	Female	3	Spayed	15	4	Salerno (Campania)
Celia/Weimaraner	Control	Female	7	Spayed	30	5	Salerno (Campania)
Muffin/Mongrel	Case	Female	4	Spayed	19	4	Salerno (Campania)
Boris/Shepard mix-breed	Control	Male	7	Intact	37	5	Napoli (Campania)
Liam/Hunting mix-breed	Control	Male	5	Neutered	28	4	Napoli (Campania)
Buddy/Mongrel	Control	Male	6	Intact	22	4	Napoli (Campania)
Heidi/Bobtail	Control	Male	7	Neutered	50	5	Napoli (Campania)

The animals underwent a complete blood test, which also included the evaluation of thyroid function, and protein electrophoresis, to rule out any co-morbidities, potentially related to behavioral issues [53, 54]. A small patch of hair was shaved from the dog’s neck and topical anaesthesia (Eutectic Mixture of Local Anaesthetics (EMLA™) cream) applied to the area before collection of a 5 ml blood sample from the jugular vein. Blood and stool samples were collected from each animal between 7:00 and 10:00 am, immediately frozen on dry ice, and stored at -80°C until their further processing. A part of stool samples was sent to a laboratory for a coprological examination, aiming at identifying the presence of intestinal parasites, to avoid any potential interference with the gut microbiota. The laboratory parameters assessed in the dogs were all in the ranges. The blood sample was collected during routine veterinary examinations, and as part of canine health screening; therefore, this research was performed without any further suffering or discomfort to the animals. All methods are reported in accordance with Animal Research: Reporting of In Vivo Experiments (ARRIVE) guidelines [55].

### Faecal bacterial community profiling

Genomic DNA was extracted from each collected faecal sample by using the RSC Blood DNA kit and was purified using the Maxwell RSC instrument (both from Promega, Madison, WI, USA). In detail, 100 mg of each sample were treated with 400 μL of lysis buffer and vortexed in order to completely homogenize it. After an incubation at 95°C in a thermomixer for 5 min at 800 rpm and a centrifugation step at 13,000 rpm for 5 min, 300 μL of supernatant were transferred into a new tube. Subsequentially, 30 μL of proteinase K were added, the samples were vortexed and incubated at 56°C in thermomixer for 20 min at 500 rpm. Then, the samples are loaded to the RSC cartridge to complete the extraction and eluted in 100 μL of elution buffer. Genomic DNAs concentration and quality were analyzed using a Nanodrop spectrophotometer (Thermo Fisher Scientific, Waltham, MA, USA). Starting from the DNA extraction, two blank/ negative samples were also included as controls and processed together with the dog’s samples during each analytical step to check for any potential environmental contamination.

### Illumina amplicon sequencing and bioinformatic analysis

For the microbiome composition analysis, 16S rRNA custom primers able to selectively amplify the V4-V6 hypervariable regions were used. To this end, a first-round PCR was carried out paying attention to optimize PCR mix and amplification conditions in order to avoid non-specific products and/or primer-dimer formation [56, 57]. AmpliTaq Gold polymerase, GC enhancer (both from Thermo Fisher Scientific, Waltham, MA, USA) and 20 μM of forward and reverse custom primers were used. Then, all PCR products were quality analysed through a 2% agarose gel and purified by using AMPure XP beads (Beckman Coulter, Brea, CA, USA). After a quality-check analysis on the Tape Station System with the D1000 ScreenTapes (both from Agilent Technologies, Santa Clara, CA, USA), the purified amplicons were quantified with Qubit HS (Qubit, dsDNA HS Assay, Life Technologies, Carlsbad, CA, USA) and diluted to 2 ng/μL to be further processed for the second-round PCR. During this step, Nextera DNA CD Indexes (Illumina, San Diego, CA, USA) were used to specifically tag each sample, and also to add the universal adapters for the following NGS reactions. Next, further AMpure XP beads-based purification and Tape Station qualitative analysis were carried out. Finally, each sample, together with the six negative controls, were quantified with the Qubit fluorometer (Life Technologies) and diluted at 4 nM. Five μL of each diluted library and 5 μL of each negative control were pooled at equimolar concentrations. MiSeq reagent Kit V2.5 500 cycles (250X2) on the MiSeq instrument (Illumina, San Diego, CA, USA) was used for the sequencing reaction. The library’s pool was loaded at a final concentration of 9 pM with a 30% PhiX.

The FASTQ files produced by the sequencing run were subsequently analyzed by the CEINGE—Biotecnologie Avanzate Franco Salvatore Bioinformatic Facility (Naples, Italy). A first quality check analysis was conducted by using FastQC software. Next, the alignment against the reference database SILVA NR 99 v.138 enabled to the correct assignment of the OTUs (Operational Taxonomic Units). The obtained OTU and taxonomy tables were used as input files for the web-based tool Microbiome Analyst (version 2.0, last accession September 2023), that allowed for the deeper analysis of the bacterial community composition [58]. In particular, α diversity was evaluated applying different metrics to assess both richness and evenness; the ANOVA test was applied to verify statistically significant differences. Unweighted and weighted UniFrac distance measures were also analyzed by using the PERMANOVA test to highlight significant differences in the β diversity. Differential abundance analysis was performed using a univariate statistical test based on the EdgeR algorithm; p-values were adjusted using the FDR method [58].

Raw 16S rRNA sequence data were submitted to Sequence Read Archive (SRA) under BioProject accession number ID #PRJNA1181839.

### Serum metabolome analysis

Metabolites were identified and quantified from collected dogs’ blood serum by liquid chromatography-tandem mass spectrometry (LC-MS/MS), using a targeted metabolomics approach [59]. In detail, 10 μL of serum were transferred onto a 96-well plate containing the positions for blanks, phosphate buffer saline (PBS), calibrants, and quality controls (QC) according to the protocols of MxP^®^ Quant 500 kit (Biocrates Life Sciences AG, Innsbruck, Austria) [60]. The mixtures were dried under N_2_, incubated for 1 h in 5% phenyl isothiocyanate (PITC) and then added with 5 mM ammonium acetate in methanol to finally extract metabolites by plate centrifugation. LC-MS/MS analysis of metabolomes was carried out in multiple reaction monitoring (MRM) mode using a Triple Quad 5500+ QTRAP^®^ Ready (AB Sciex, Framingham, MA, USA) coupled to a 1260 Infinity II HPLC (Agilent, Santa Clara, CA, USA). Each sample was run by LC-MS/MS-MRM three times as technical replicates. Raw MS data were processed with the Analyst software v.1.7.1 (AB Sciex) and the MetIDQ^TM^ Oxygen software (Biocrates Life Sciences AG) to integrate targeted metabolite peaks for accurate quantification with respect to labelled internal standards, and finally expressed as μM. The metabolomic analysis allowed to target 106 small molecules, including amino acids (AA) and related AA, bile acids, fatty acids, biogenic amines, carboxylic acids, hormones, indoles derivatives, alkaloids, amine oxides, cresols, vitamins, and cofactors.

The metabolomic dataset was first processed using the MetaboAnalyst 5.0 tool for chemometrics and statistical analyses [61, 62]. The features with more than 50% of missing values were removed, and missing values were imputed using 1/5 of minimum positive values of their corresponding variables. The dataset was normalized, log10-transformed and auto-scaled. Partial Least Squares-Discriminant Analysis (PLS-DA) was performed to find the variance between the (case and control) groups and predict the class of relevant features according to the VIP (Variable Importance in Projection) score. Metabolites with VIP score>1.5 were selected as predictive of the phenotypes analyzed. Hierarchical clustering analysis and heatmap visualization were performed to check intra- and inter-group biological variability according to the relative abundance of their metabolite features. The technical triplicates of each sample were kept as individual features (not averaged) for PLS-DA and heatmap clustering also to assess the reproducibility of metabolomics data. MetaboAnalyst was also used for pathway analysis and Metabolite Set Enrichment Analysis (MSEA) through the Over Representation Analysis (ORA) function [63]. For pathway enrichment analysis, the Homo sapiens KEGG was chosen as pathway library in absence of a proper database relative to dog species; matched pathways were selected according to the p-values from the pathway enrichment analysis and pathway impact values from the pathway topology analysis (i.e. the selected pathway showed concurrently -log(p)>1.3 and pathway impact>0.1). For the enrichment of ORA terms, significant terms were selected for values of p<0.05.

### Metabolome data

The normalized dataset was exported from MetaboAnalyst to execute univariate statistical analysis within GraphPad Prism 9.0 software [64]. In particular, volcano plot analysis was carried out to select metabolites with significantly changing abundance. Individual log2 abundance values were averaged within each group, and the fold change was calculated as difference of the metabolite levels in fearful and control dogs.

The significance threshold was set at p<0.01. On the other hand, the significant differences for binary comparisons (between cases and controls) were evaluated for the single molecules, i.e. those significant from volcano plot analysis, using the non-normalized concentration (μM) dataset obtained by LC-MS/MS. Parametric Welch’s t-test or non-parametric Mann-Whitney t-test were used as statistical tests according to the normality of the distributions, assessed by D’Agostino & Pearson test. Finally, correlation analysis between significant microbiota species and metabolites was performed computing Spearman correlation and simple linear regression.

Metabolomics data have been deposited to the EMBL-EBI MetaboLights database (DOI: 10.1093/nar/gkad1045, PMID:37971328) with the identifier MTBLS11669.

## Results

**Fig 1:**
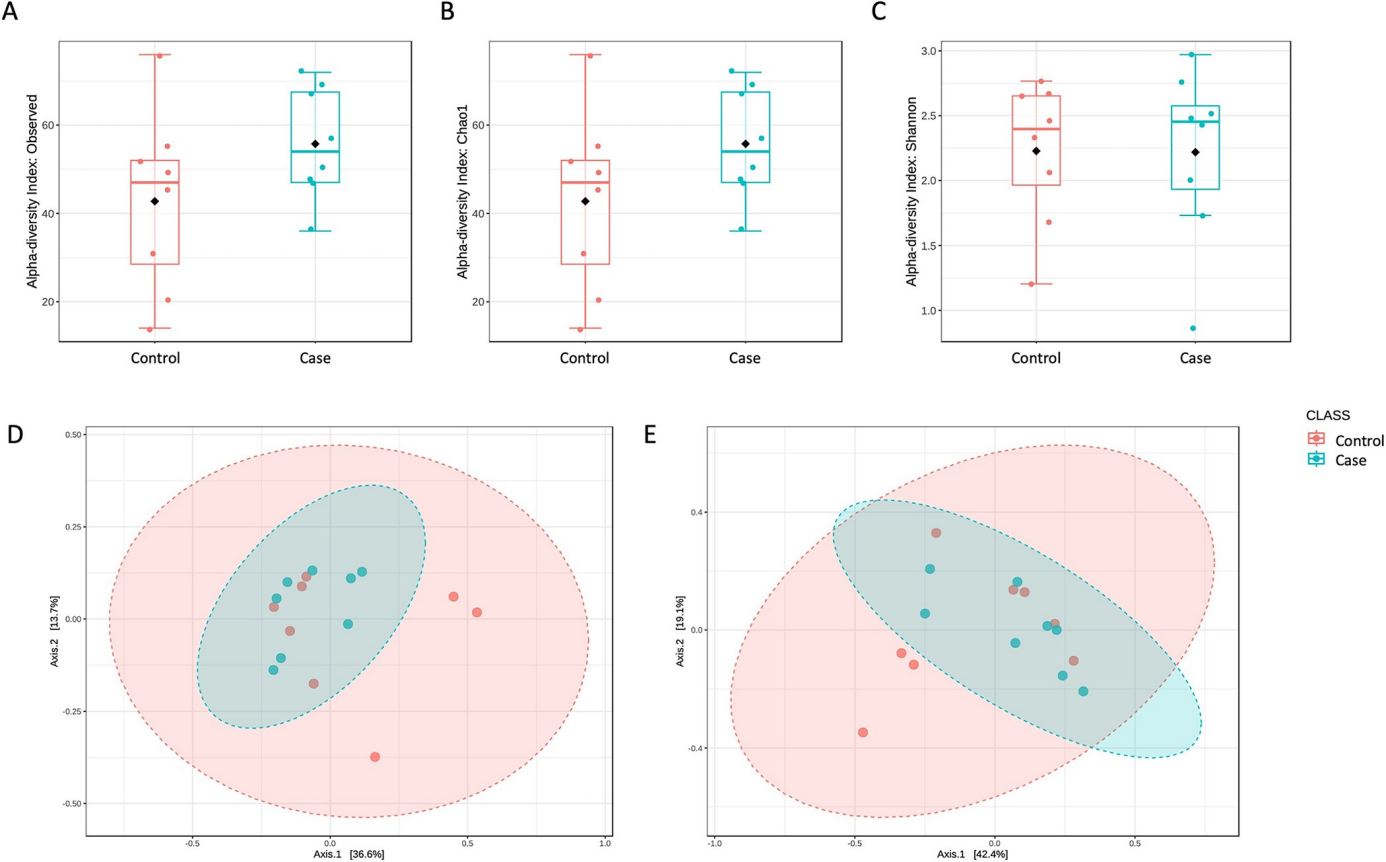
Diversity measures between the fearful dogs (case) and the dogs with normal behavior (control). Alpha diversity was assessed by using Observed species ((A), p = 0.14), Chao1 ((B), p = 0.14) and Shannon ((C), p = 0.97), indices and the ANOVA test. Β diversity was also evaluated by using the unweighted ((D, p<0.52) and weighted ((E, p<0.2) UniFrac distance measures, applying the PERMANOVA test. No significant differences were identified by all the above-mentioned tests.

The sequencing reaction produced a total yield of 6.9 Gb, a cluster passing filter of 93.9% and a cluster density of 697 K/mm^2^. All the reads detected in at least one of the six negative controls were filtered out and eliminated from the rest of the samples in a normalization- process. At the end of this decontamination step, an average of 235,827 reads/sample, corresponding to a total of 706 OTUs, was obtained and used for subsequent analyses. First, microbial communities’ diversity measures were assessed. In particular, to verify both the richness and the evenness, alpha diversity was evaluated within two groups, and the ANOVA test was applied to highlight any significant difference. As shown in Fig 1, all the different metrics used (panel A: Observed Species, panel B: Chao1, and panel C: Shannon) showed no significant differences between groups. However, a different trend could be appreciable, richness and evenness being both higher in the case than in the control group. In addition, beta diversity analysis was carried out by using both unweighted (Fig 1D) and weighted (Fig 1E) Unifrac distance measures, and no significant differences were found.

**Fig 2:**
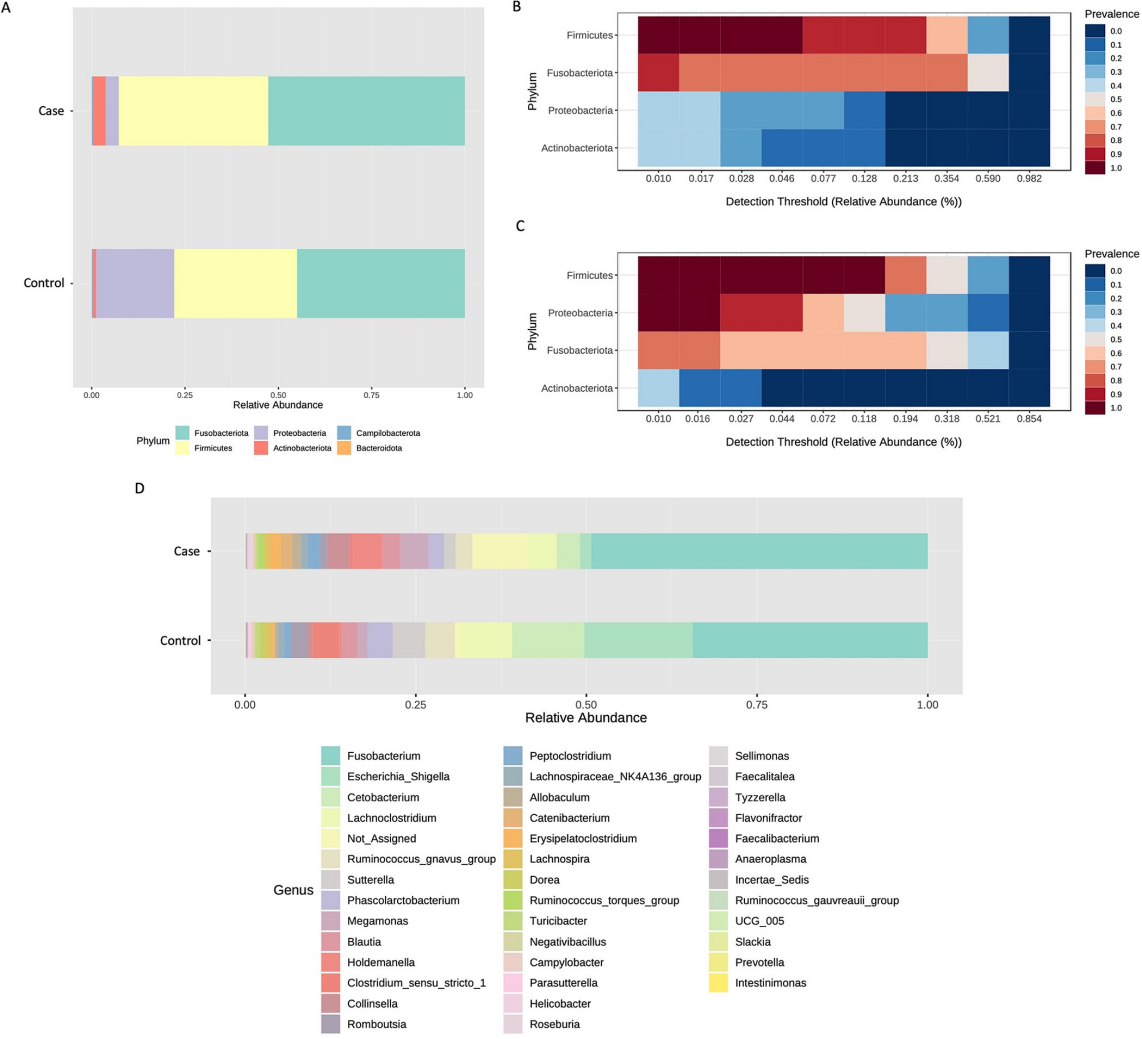
Taxonomic assignment highlighted different microbial taxa in case and control groups. A different bacterial composition (relative abundance, %) was highlighted at the phylum level (A) and confirmed by core microbiome analysis showing a different taxa profile in Case (B) and Control (C) groups. These differences in taxa composition were also identified at the genus level, as shown in panel (D).

Although the diversity measures showed no significant differences between the two tested conditions, it can be noticed that the case group showed a reduced heterogeneity compared to the control, suggesting that the disease status may be related to common microbial features. Next, taxonomy assignment allowed to identify six phyla, four of which with an abundance >1% in at least one of the 2 compared groups (Fig 2A). Among these, we found a lower relative abundance of Proteobacteria in the case vs control group (3.5% vs 20.9%), while Fusobacteriota (44.9% vs 52.7%), Firmicutes (32.9% vs 40%), and Actinobacteriota (0.8% vs 3.3%) phyla were more abundant. Accordingly, the core microbiome analysis, considering a relative abundance >1% and a 20% value of sample prevalence, showed a different set of taxa characterizing the case (Fig 2B) and the control group (Fig 2C). At genus level, out of nine most represented taxa, we found six bacteria highly expressed in the case vs control group, namely *Cetobacterium* (10.6% vs 3.44%), *Escherichia_Shigella* (from 15.9% to 1.6%), *Lachnoclostridium* (from 7.6% to 4%), *Phascolarctobacterium* (3.7% vs 2.2%), *Ruminococcus_gnavus_group* (4.4% vs 2.5%), and *Sutterella* (4.7% vs 1.7%). On the other hand, *Fusobacterium*, *Holdemanella* and *Megamonas* were found at lower levels in the case group (*Fusobacterium*, 34.4% vs 49.2%; *Holdemanella*, 0.4% vs 4.2%; *Megamonas*, 1.4% vs 4.2%) (Fig 2D).

**Fig 3:**
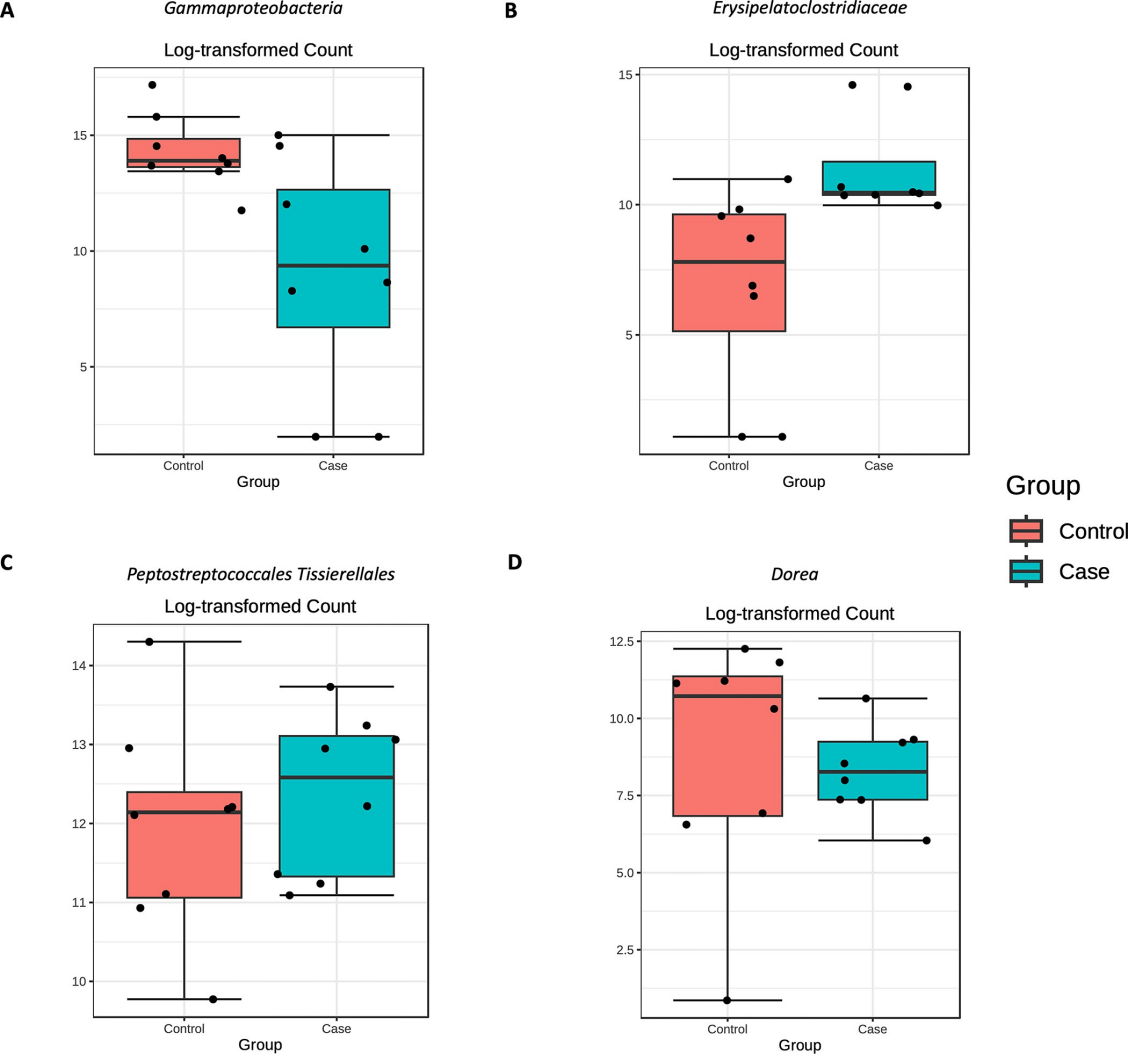
Significantly different taxa identified between case and control groups by EdgeR analysis (adjusted p-value <0.05). Among the significantly different taxa we found that the *Gammaproteobacteria* class was less abundant in the cases respect to the controls (A). The Firmicutes-related taxa, *Erysipelatoclostridiaceae* family (B) and *Peptostreptococcales Tissierellales* order (C) were more abundant in the cases, when compared the controls. At genus level, the Firmicutes-belonging *Dorea* taxon was reduced in the case group (D).

Then, to verify if these differences are statistically significant we performed a differential abundance analysis. In particular, EdgeR analysis allowed us to identify a lower abundance of *Gammaproteobacteria* and *Dorea* in fearful patients (Fig 3A and 3B), who also displayed higher levels of *Erysipelatoclostridiaceae* and *Peptostreptococcales Tissierellales* (Fig 3C and 3D), when compared to the healthy controls.

**Fig 4:**
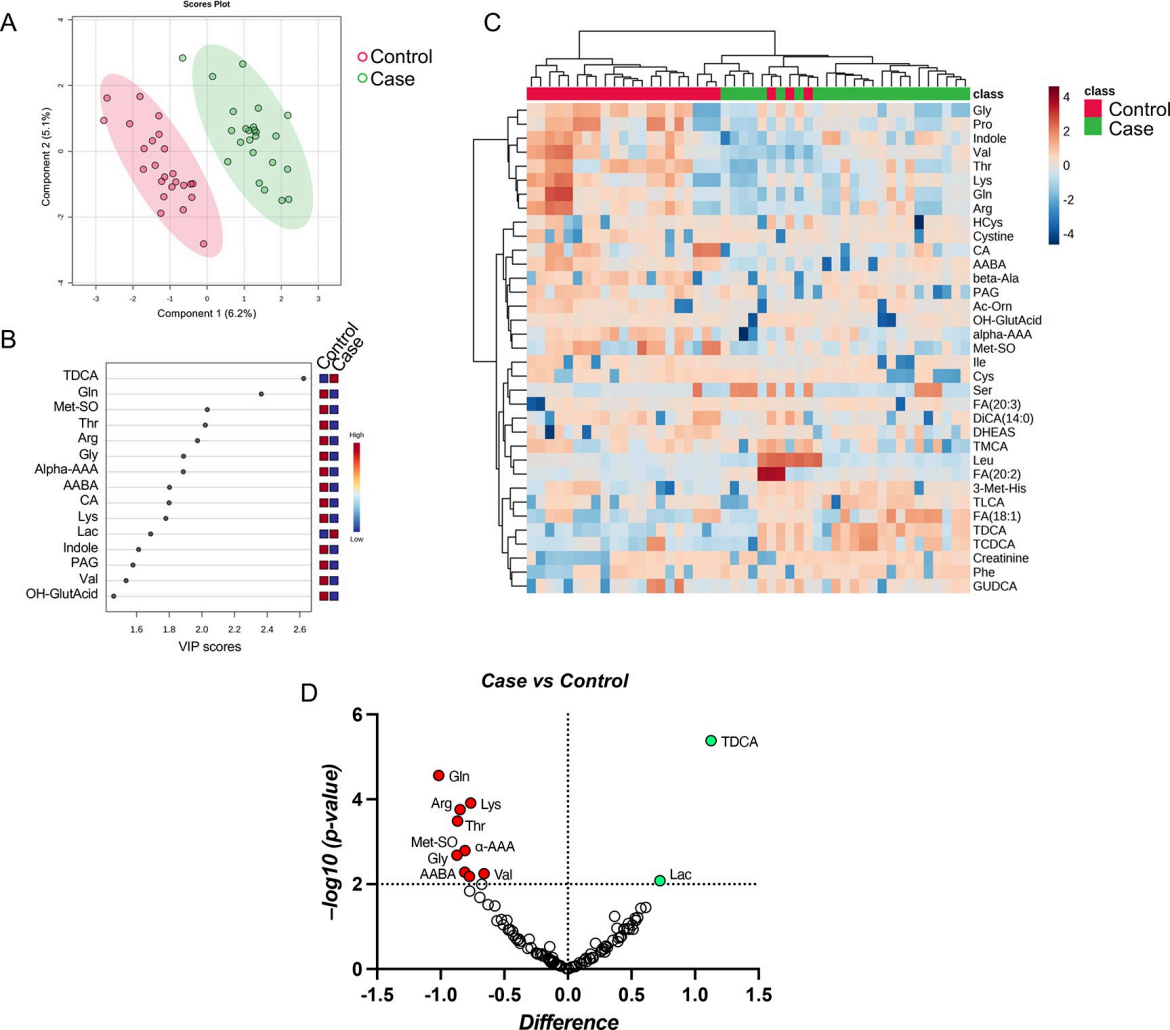
Comparative metabolome analysis of case (fearful dogs) versus controls. (A) PLS-DA analysis was performed using normalized metabolite levels from cases (green) and controls (red); (B) The top-15 discriminant features identified with values of VIP scores >1.5 are reported. (C) Heatmap showing metabolite concentrations in each group and replicates. The intensity of the colored boxes represents the relative abundance of each molecule, whereas metabolite concentrations were normalized, log(10)-transformed, and auto-scaled. (D) Volcano plot analysis of metabolites significantly different in the comparison case vs control. The red and green dots represent the significant increased and decreased metabolites, respectively. Non-colored dots refer to all the molecules identified in the dataset whose relative abundance is not significantly different between groups.

To further investigate the systemic changes affecting fearful dogs, a metabolome analysis was performed on the blood serum samples, using a targeted LC-MS/MS approach. The quantitative metabolomics dataset was included in S1 File. Univariate and multivariate statistical analyses were employed to select the most significant metabolic alterations in fearful animals. Specifically, the variances between case compared to control group revealed a good spatial separation according to the PLS-DA, with variances of principal component 1 (PC1) and 2 (PC2) of 6.2% and 5.1% (Fig 4A). The Variable Importance in Projection (VIP) score was used to identify those metabolites (VIP >1.5), taurodeoxycholic acid (TDCA) and glutamine (Gln) as examples, whose levels can strongly discriminate fearful dogs versus controls, highlighting metabolic abnormalities likely connected with the fearful phenotype (Fig 4B). Also, the hierarchical clustering of quantified metabolites reported in the heatmap displayed a distinct separation pattern between cases and controls (Fig 4C). Univariate statistics performed by volcano plot analysis highlighted eleven metabolites with differential abundance in fearful dogs (Fig 4D), many of which overlap with VIP metabolites.

Quantitatively altered metabolites are listed in Table 2 in the order from the least abundant to the most abundant, along with their corresponding P-value,–log10(P-value), difference, standard error (SE) of difference, and regulation trend.

**Table 2:** Significant metabolites found differentially abundant in fearful dogs versus controls.

Metabolite	P-value	–log10(P-value)	Difference	SE of difference	Regulation
Glutamine (Gln)	0.000028	4.561	-1.015	0.1947	Down
Glycine (Gly)	0.00523	2.281	-0.8107	0.2628	Down
α-Aminobutyric acid (AABA)	0.006575	2.182	-0.7736	0.2589	Down
α-Aminoadipic acid (α-AAA)	0.00161	2.793	-0.8099	0.2266	Down
Lysine (Lys)	0.000123	3.91	-0.764	0.1657	Down
Methionine sulfoxide (Met-SO)	0.002083	2.681	-0.873	0.2517	Down
Threonine (Thr)	0.000327	3.486	-0.8679	0.2057	Down
Arginine (Arg)	0.000174	3.759	-0.8473	0.1895	Down
Valine (Val)	0.005669	2.247	-0.6594	0.2161	Down
Lactic acid (Lac)	0.008264	2.083	0.724	0.2505	Up
Taurodeoxycholic acid (TDCA)	0.000004	5.383	1.127	0.188	Up

**Fig 5:**
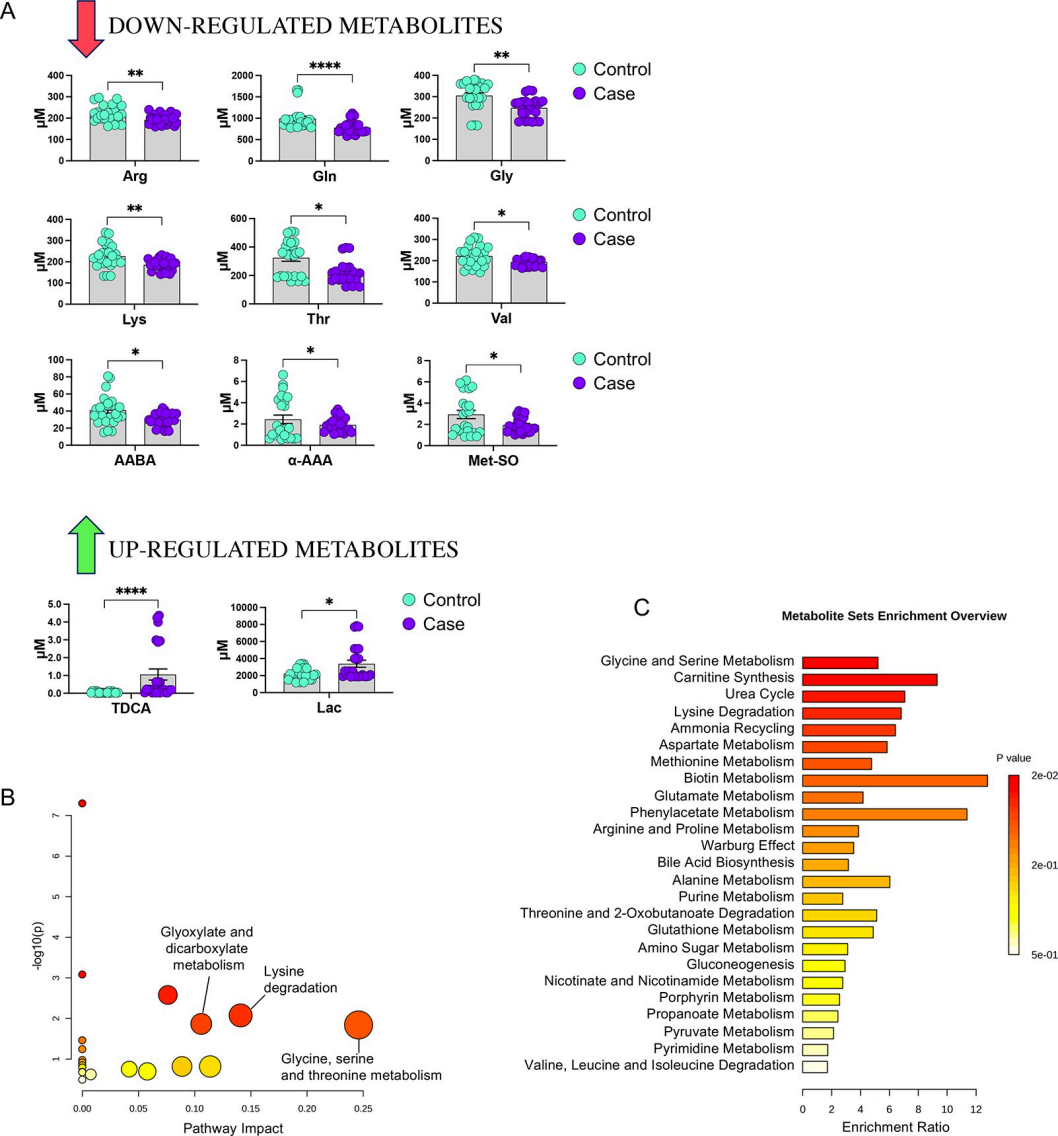
Statistical analysis and bioinformatic pathway enrichment of single metabolites from the differential metabolome. (A) The abundances of single metabolites were evaluated in case vs control by parametric or non-parametric t-test. The plots report the analyte concentrations (means ± SEM on the bar, dots are all the measurements in replicates). The significance of statistical analysis is referred to as: * p<0.05, ** p<0.01, **** p<0.0001). (B) Pathway analysis plot with details of the significant pathways enriched by the analysis. (C) Overview of MSEA terms enriched with the over representation analysis (ORA) of significant metabolites. Nonetheless, to assess whether faecal species and circulating metabolites varied with the same trends in fearful animals, we performed a correlation analysis using both types of features. Significant microbiota species were correlated with the global metabolome, highlighting significant correlations (Fig 6). Interestingly, bile acids (namely TDCA, GUDCA, GLCA) were positively correlated with the *Peptostreptococcales Tissierellales* (Fig 6A), whereas some amino acids (Val, Thr, Lys) were associated to the *Dorea* (Fig 6B). Remarkably, many of these molecules were found as significant from both volcano plot and VIP analyses.

To further validate the abundance trends in each sample group for the selected metabolites, we analyzed them individually using raw concentrations from the LC-MS/MS dataset, confirming the trends of regulation and statistical significance (Fig 5A). Additionally, we performed pathway analysis on significant metabolites to enrich dysregulated pathways associated to the fearful phenotype. Among them, we selected those with concurrently highest impact and lowest p-value, namely ‘glycine, serine and threonine metabolism’, ‘lysine degradation’, ‘glyoxylate and dicarboxylate metabolism’ (Fig 5B). MSEA performed through ORA analysis revealed biologically meaningful terms associated with the differential metabolome of fearful dogs. ORA analysis enriched as significant (p<0.05) terms: ‘glycine and serine metabolism’, ‘carnitine synthesis’, ‘urea cycle’, ‘lysine degradation’, ‘ammonia recycling’, and ‘aspartate metabolism’ (Fig 5C).

**Fig 6:**
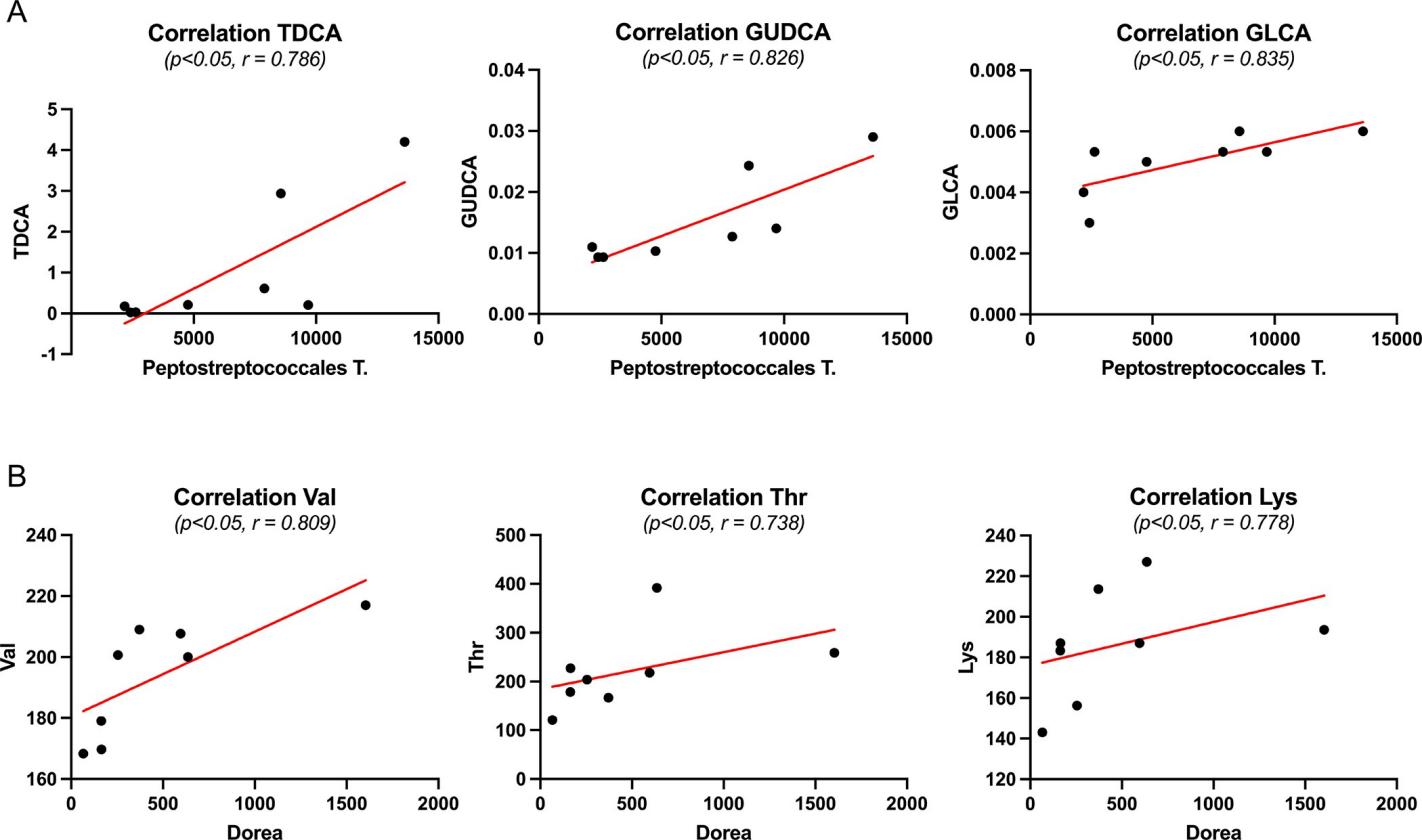
Correlation analysis of the differential microbiome with the global metabolome. Significantly correlating metabolites were reported for (A) *Peptostreptococcales T* and (B) *Dorea* organisms. The correlation plots report the computed r coefficient and the statistical significance (p). TDCA: Taurodeoxycholic acid, GUDCA: Glycoursodeoxycholic acid, GLCA: Glycolithocholic acid, Val: Valine, Thr: Threonine, Lys: Lysine.

## Discussion

In the present work, we documented the differential abundance of Proteobacteria and Firmicutes Phyla in fearful dogs suffering from generalized fear. Like in humans, companion animals diagnosed with such a behavioral disorder very often experience unreasonable, intense, and even excessive fright towards tangible stimuli or situations. These reactions can eventually trigger immediate responses and concomitant autonomic arousal, turning into escape, avoidance, or defensiveness behaviors [65]. Despite the huge amount of driving factors, dogs can also display fearful behaviors due to the lack of socialization during their early developmental phase (3–12 weeks of age), aversive experiences, as well as pharmacologic treatments or genetic susceptibility [66–70]. Furthermore, fear can lead to anxiety without the dissolution of fear itself, so that both may coexist in the same individual [71]. Tailored therapies, which generally rely on integrated approaches (drugs and behavioral treatment), are employed to reduce excessive fear or/ and anxiety reactions, by fostering positive and emotional situations, and avoiding punishment or correction during training activities [71, 72]. Indeed, dogs who suffer from generalized fear and anxiety generally require to be pharmacologically treated with selective serotonin reuptake inhibitors (SSRIs) or benzodiazepines, that allow them to cope with such a dysfunctional situation. Unfortunately, the owners’ resistance in using medications, mostly associated with the occurrence of their side effects, including drowsiness, paradoxical arousal, vomiting, diarrhea, and restlessness, sometimes make pharmacological approach less feasible. Therefore, more thoughtful research, characterized by the development of new strategies to counteract mood-related dysfunctions is a way forward [73]. Compelling and growing evidence in mammals highlighted the crucial role of the microbiota, one of the main physiologic players within the gastrointestinal tract [74]. Previous studies found that germ-free mice exhibited alterations in immune systems, hormone signalling, metabolism, neurotransmission and synaptic plasticity, impacting upon physiologic homeostasis and development [74, 75]. Moreover, GI microbiota-depleted young mice, transplanted with the stool of the aged animals, showed age-dependent spatial learning and memory deficits, without affecting the short-term cognitive ability [76]. Thus, the overall intestinal microbiota has been regarded as one of the main peripheral modulators of CNS physiology, either by synapsing with vagus nerve, or passing through the blood brain barrier (BBB). The composition and integrity of the BBB can be affected by a dysfunctional GI microbiota, which makes it more permeable, most likely due to the reduced expression of tight junctions [77]. This altered permeability causes toxins to enter the brain, thus affecting the release of neurotransmitters, like serotonin, dopamine, GABA, and eventually bringing about mood-related disorders [78–81]. Our results documented an increase of Firmicutes-related taxa in the fearful dogs, being in line with previous findings, about an increase of the Firmicutes-related *Lactobacilli* in animals with emotional discomfort [20, 51]. Again, these aerotolerant anaerobic microbes increased in rescued dogs, who experienced intraspecific aggression [50, 82]. Studies from Mondo and colleagues (2020), showed that *Lactobacilli* were significantly higher in a group of German shepherds, who displayed a similar GI microbiota to that of depressed humans [83]. Moreover, *Lactobacillus Rhamnosus* was also found to reduce stress-associated corticosterone levels and anxiety-related behaviors and affect GABA-dependent neurotransmission in mice [84]. Taken together, it is conceivable that *Bacilli* could modulate several aspects of the microbiota-gut-brain axis, although care must be taken in extrapolating data, since identifying the precise mechanism underlying the observed increase of *Lactobacillus* in fearful dogs it not always immediate. Some anaerobic microbes belonging to Bacteroidetes and Firmicutes phyla generate butyrate, propionate and acetate as by-products of the indigestible polysaccharides’ fermentation. These short-chain fatty acids (SCFAs), once crossing BBB, can affect cognition and mood-related behaviors [85]. According to our data, about a significant reduction of *Dorea* taxon in fearful dogs, lower levels of this Firmicutes-belonging genus were also previously found in humans and animals suffering from behavioral disorders [50, 86, 87]. Together with *Akkermansia* and *Ruminococcus*, *Dorea* abudance correlated with anxiety and depressive-like behavior in mice exposed to social defeat stress protocol, suggesting a potential role for such bacteria in the gut-brain axis communication [20, 88]. We found a direct correlation between gut *Dorea* abundance and serum levels of valine, threonine and lysine metabolites, which might represent metabolic substrates for SCFAs production [89].

The higher levels of the Firmicutes-related taxa found in our patients well fit with serum levels of lactic acid in fearful dogs, since they produce lactic acid, resulting in acidification of environment that can inhibit the growth of some pathogenic microorganisms [90]. It is well recognized that Firmicutes are involved in the conversion process of primary to secondary bile acids (and then deconjugating all the glyco- and tauro-conjugated forms) [91, 92], thereby hesitating in an antimicrobial function, which is more powerful than primary cholic acid, owing to its ability to damage microbial membranes. Accordingly, we found that *Peptostreptococcales Tissierellales* order, belonging to the Firmicutes phylum, positively correlated with TDCA, GUDCA and GLCA metabolites. Future studies, aimed at better disclosing the role of *Peptostreptococcales Tissierellales* in the gut-brain axis, are required. Our metabolomic evaluations reported the downregulation of glycine in the blood of fearful patients which, together with glutamine and α-Aminobutyric acid, can modulate GABA-dependent neurotransmission, and impact several psychiatric disorders in humans [93, 94]. We also found a significant reduction of serum α-Aminoadipic acid levels that, as an intermediary metabolite of lysine and tryptophan, has been proven to antagonize neuroexcitatory activity modulated by the glutamate N-methyl-D-aspartate receptor [95].

Dysregulation of the GI microbiota, characterized by a relative increment of Proteobacteria, has been documented in IBS, metabolic and inflammatory disorders, so that these gram-negative microorganisms and their related taxa can be regarded as potential markers of microbiota instability [96–98]. The differential and quantitative analysis showed a lower abundance of Proteobacteria-related *Gammaproteobacteria* in the case group, allowing us to infer a putative compensatory effect, which comes into play to avoid the worsening of behavioral symptoms. However, more additional studies involving larger number of patients are needed to support this hypothesis.

On the other hand, a potential gender effect underlying the reduction of *Gammaproteobacteria* population in the case group (predominantly females) should be considered, since evidence in humans showed that Proteobacteria were more abundant in males than females [99]. Moreover, the higher incidence of female dogs suffering from fear and anxiety might be in accordance with human findings, showing that women are likely to double anxiety-related disorder chances [100].

Even though the sample size is small, which represents one of the main concerns of the manuscript, in the present study we involved individuals from different cities and fed with an overlapping dry commercial diet, to reduce nutritional and environmental variability. At present, it is hard to define whether alterations in the microbiota are the trigger or the consequence of behavioural changes. However, it is likely that both scenarios coexist in a vicious circle, with the initial trigger occurring both centrally and peripherally.

## Conclusions

Our data emphasized for the first time how fear and anxiety might be linked to significant dysregulation in both GI microbiota and blood metabolic homeostasis of family dogs affected by generalized fear. Despite recent advances in veterinary medicine, further investigation pointing towards multidisciplinary approaches are required, to better understand the pathophysiology of behavioral dysfunctions, and develop new pharmacological targets for canine mood-related disorders.

### Limitations

We recognize that our study involved a small number of patients, due to the tightening factors we have chosen as inclusion criteria in the recruitment and in the analyses performed. Future studies are mandatory to disentangle the role of the gut-brain axis homeostasis in the modulation of behavioral dysfunctions, enrolling a larger population of dogs, homogenously clustered for environment, sex, size, lifestyles, and genetics.

## Supporting information

S1 File (ZIP)
